# Comparison the effect of putrescine application on postharvest quality of *Pyrus communis* cv. “Shah‐Miveh” and “Spadona”

**DOI:** 10.1002/fsn3.764

**Published:** 2018-11-08

**Authors:** Marjan Sadat Hosseini, Mesbah Babalar, Mohammad Ali Askari, Seyed Morteza Zahedi

**Affiliations:** ^1^ Department of Horticulture Science Faculty of Agriculture and Natural Resources University of Tehran Karaj Iran; ^2^ Department of Horticultural Science Faculty of Agriculture University of Maragheh Maragheh Iran

**Keywords:** ascorbic acid, color, firmness, pear, weight loss

## Abstract

Role of putrescine for extending storage life of pear cv. “Shah‐Miveh” and “Spadona” was evaluated. The trees were sprayed by various concentrations of putrescine (0.5, 1, and 2 mM) and distilled water “control.” After harvest, all samples were stored at 0 ± 1°C, 80%–85% RH for 21 weeks. Thereafter, some physico‐chemical attributes were measured initially and after each storage period 7, 14, and 21 weeks. Putrescine at 1 and 2 mM reduced fruit softening, weight loss, color changes (L*, hue angle), fungal infection as well as retarded the degradation of total soluble solids, titratable acidity, ascorbic acid, total phenol (TP), and total antioxidant activity (TAA). However, fruit softening, weight loss, and hue angle rates were slower in “Shah‐Miveh” to “Spadona.” Moreover, at the end of storage, “Shah‐Miveh” demonstrated more TP and TAA in compare to “Spadona.” Thus, putrescine application at higher values may be an effective tool to prolong pear postharvest life during storage.

## 1 INTRODUCTION

Pears are considered a valuable source of natural antioxidant due to the presence of “miscellaneous phenolic compounds” (Galvis‐Sanchez, Gil‐Izquierdo, & Gil, 2003). *Pyrus communis* is a climacteric fruit; therefore the rate of ripening process is associated with ethylene synthesis. As the pear softens during the ripening process, storage life and shelf life (SL) decreases. As a pear is a perishable fruit it will become ripe quickly in a short period of time. Therefore, the application of postharvest treatment would be crucial to extend SL and maintain high quality of the fruit. Nowadays, the application of chemical synthetic fungicides on the fruit has been restricted due to their harmful effects on human health and environment. Thus, there is a demand for further studies seeking alternative methods to improve storage life of fruits.

Polyamines are aliphatic with low molecular weight organic compounds having 3–15 linear carbon and two terminus amino groups (Kalac & Krausov, 2005). These compounds link to the membrane and make it tighter and subsequently change the membrane permeability and cause active transportation from it (Tiburcio et al., 1994). Polyamines affect enzymes activity by direct effect on cross‐linked enzymes or with effect on minus charges on the surface of the membrane. Exogenous application of polyamines makes the plasma membrane more stable and prohibits decomposition of chlorophyll. Moreover, these compounds increase the synthesis of DNA, RNA, and proteins (Lee, Lee, & Park, 1997). The polyamines inhibit the ethylene production by regulating the activity of synthase and oxidase of 1‐aminocyclo‐propane‐1‐carboxylic acid (Lee et al., 1997), while ethylene alters the polyamines formation by reducing the arginine decarboxylase activity and SAM decarboxylase (Roustan, Chraibi, Latche, & Fallot, 1993).

It is reported that polyamines had antiaging effects on honeydew muskmelon fruit and exogenous application of these compounds prolonged the SL of fruits by retaining firmness, reducing ethylene production, delaying color change, total soluble solids (TSS) and titratable acidity (TA) as well as protecting fruits against chilling injury and mechanical damages (Lester, 2000; Valero, Martinez‐Romero, & Serrano, 2002). Application of polyamines in peach fruits reduced the production of ethylene, TSS, and pH, while increased the TA, fruit firmness and prolonged SL (Zokaee Khosroshahi & Esna‐Ashari, 2008).

The application of polyamines increased postharvest life of fruits by reduction in ethylene production, TSS, acidity of peach (Zokaee Khosroshahi & Esna‐Ashari, 2008), plum (Khan, Singh, Abbasi, & Swinny, 2008), reduction in flavor index in nectarine (Torrigiani et al., 2004). Moreover, polyamines had positive impacts on retarding fruit softening in peach (Bregoli et al., 2002; Martinez‐Romero et al., 2000), pear (Franco‐Mora, Tanabe, Tamura, & Itai, 2005), color change delaying in kiwifruit (Petkou, Pritsa, & Sfakiotakis, 2004), lemon (Valero, Martinez‐Romero, Serrano, & Riquelme, 1998), and decrease fungal contamination in grapes (Mirdehghan & Rahimi, 2016).

Therefore, the objective of this investigation was to assay pre‐ and postharvest application of putrescine in maintenance of quality and prolonged the storage life of pear cv. “Shah‐Miveh” and “Spadona.”

## 2 MATERIALS AND METHODS

### 2.1 Plant material and treatments

The experiment was carried out on pear trees (*P. communis* cv. “Shah‐Miveh,” “Spadona”) in research center of horticulture of the University of Tehran, Karaj, Iran. Eighteen trees (16‐year‐old) for “Shah‐Miveh” and eighteen trees for “Spadona” fruits were selected for uniform size and fruit load, and used for treatments by spraying putrescine at different concentrations (0.5, 1 and 2 mM) three times during the fruit development in May, June, and July. The full bloom stage in Karaj condition was in late of April, and the first spraying stage was a month later. Six trees only received water as control. Experimental layout was randomized block design and two trees in each experimental unit with three replications.

Fruits were harvested manually at green maturity stage based on number of days after full bloom. Afterward, they were transported to the postharvest laboratory and selected for the absence of visual symptoms of disease or blemishes, then stored (five fruits per basket) at 0 ± 1°C and 80%–85% relative humidity for 21 weeks.

Three replicate of individual treatment were taken at different time intervals including: at harvest (day 0) and at weeks 7, 14 and 21 after storage and with one additional day of keeping at 20°C as SL.

### 2.2 Weight loss and firmness

Weight loss was determined on the day of harvesting and after the different sampling dates. Cumulative weight losses were expressed as percentage loss of original weight.

Flesh firmness was measured using a fruit penetrometer (Model FT 327, Italy) with an 8 mm diameter tip. A small slice of fruit skin was removed and firmness was recorded from both sides of individual fruit. Means were expressed as Newton (N).

### 2.3 Total soluble solids, TA, and flavor index

The TSS of fruit juice was determined using digital refractometer (Atago N1, Japan) and was expressed as percentage. Five milliliters of diluted juice titrated against 0.1 M NaOH to pH 8.2. To determine TA, Phenolphthalein was used as an indicator. The TA was expressed as percentage malic acid. Flavor index was calculated by dividing TSS with corresponding TA value.

### 2.4 Color changes

Color change was determined at three of sides of each fruit from each replicate with a Minolta Chromameter CR400 in which the L* (0—black; 100—white), a* (green to red) and b* (blue to yellow) then expressed L* and hue angle (*h*°) = 180 + tan^−1^ b*/a*, if a* < 0 (Fernando, Wang, Wang, & Gonzalez, 2007; Pek, Helyes, & Lugasi, 2010).

### 2.5 Ascorbic acid content

The ascorbic acid concentration in fruit extracts was determined by titration using a solution containing I and KI (16 g KI and 1.72 g I in 1 L water). The titration ended when the sample turned dark blue and color was stable for a few seconds. The volume of the I + KI solution was recorded and the concentration of ascorbic acid calculated according to the following equation (O'Grady, Sigge, Caleb, & Opara, 2014) as ([0.88 × *V*]/5) × 100), where *V* is the volume of the consumed I + KI solution.

### 2.6 Total phenol and total antioxidant activity

One gram of frozen sample (in liquid nitrogen and kept at −80°C) from each replicate was homogenized in 5 ml of ice‐cold 1% HCl‐methanol solution and centrifuged at 16000 × *g* at 4°C for 10 min. The supernatant was used for total phenol (TP) analysis. The TP was determined according to the Folin–Ciocalteu method (Serrano, Guillen, Martinez‐Romero, Castillo, & Valero, 2005) and gallic acid was applied as a standard curve. Results were expressed as milligrams of gallic acid equivalent per 100 g of fresh weight. Total antioxidant activity (TAA) was assessed as described by Koushesh Saba, Arzani, and Mohsen Barzegar (2012).

### 2.7 Microbial analysis

Microbial analysis was measured by homogenizing 10 g of sample from each replicate with 90 ml of sterile peptone water for 90 s using a stomacher blender (Bag Mixer 400; InterScience, France). Serial dilutions of fruit homogenates were prepared and 1 ml of final preparation was poured in plate count agar under sterile conditions (Valero et al., 2005). All plates were incubated for 5 days at 25°C and counts of colony forming were evaluated. Results were expressed as Log CFU/g.

### 2.8 Statistical analysis

The experimental data were subjected to analysis of variance (ANOVA) using SAS (V 9.3, SAS Institute Inc.) package and the means were compared by Duncan's test. Sources of variation were: storage time, treatment, fruit cultivars, and block.

## 3 RESULTS AND DISCUSSION

### 3.1 Weight loss and fruit firmness

**Figure 1:**
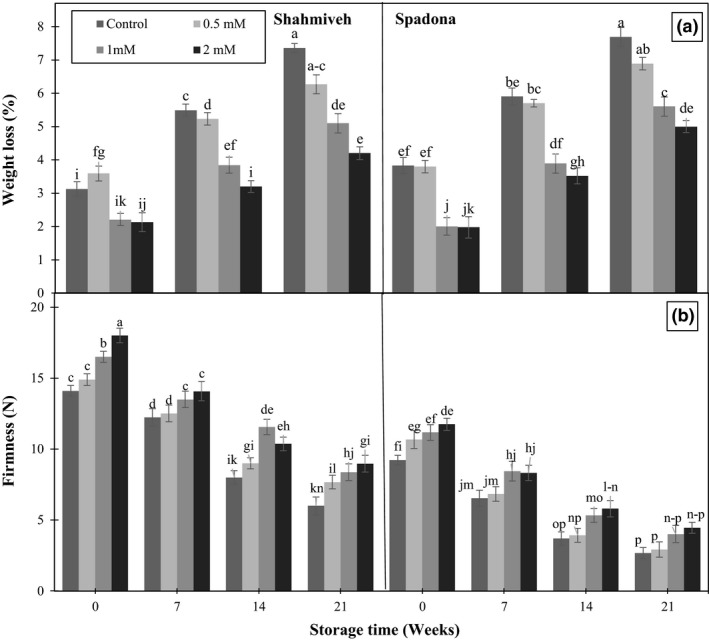
Effect of putrescine on weight loss (a) and firmness (b) of pear cultivars of “Shah‐Miveh” and “Spadona” stored at 0 ± 1°C. Data are means ± *SE*. Values with similar letters did not show significant difference (*p* < 0.05)

The cumulative weight loss increased during storage irrespective of treatment in both cultivars (Figure 1a). However, it was significantly higher in control on the contrary to putrescine‐treated pears. Significant lower weight loss of pears was observed in “Shah‐Miveh” than “Spadona.” The lower weight loss in putrescine‐treated fruit may be ascribed to stabilization of cell membrane integrity (Enas, Sarrwy, & Hassan, 2010; Mirdehghan et al., 2007). These results are in accordance to previous works (Enas et al., 2010; Zokaee Khosroshahi & Esna‐Ashari, 2008).

Fruit firmness markedly declined regardless of treatment with the ripening advancement during storage. However, higher fruit firmness was observed in “Shah‐Miveh” as compared to the “Spadona.” In both cultivars, the highest levels of firmness were observed in purescine 1 and 2 mM at harvest and after storage time than control (Figure 1b). A delay in fruit softening and subsequently fruit firmness could be attributed to the cross‐linkage of putrescine with carboxyl (COO^−^) group of the pectic substances in the cell wall, resulting in wall stability and consequently reducing the activities of cell wall degrading enzymes including pectin methyl esterase, pectinesterase and polygalacturonase (Valero et al., 2002). The results are in agreement with those in mango (Malik & Singh, 2005), plum (Khan et al., 2008) and pear (Franco‐Mora et al., 2005).

### 3.2 Total soluble solids, TA, and flavor index

**Figure 2:**
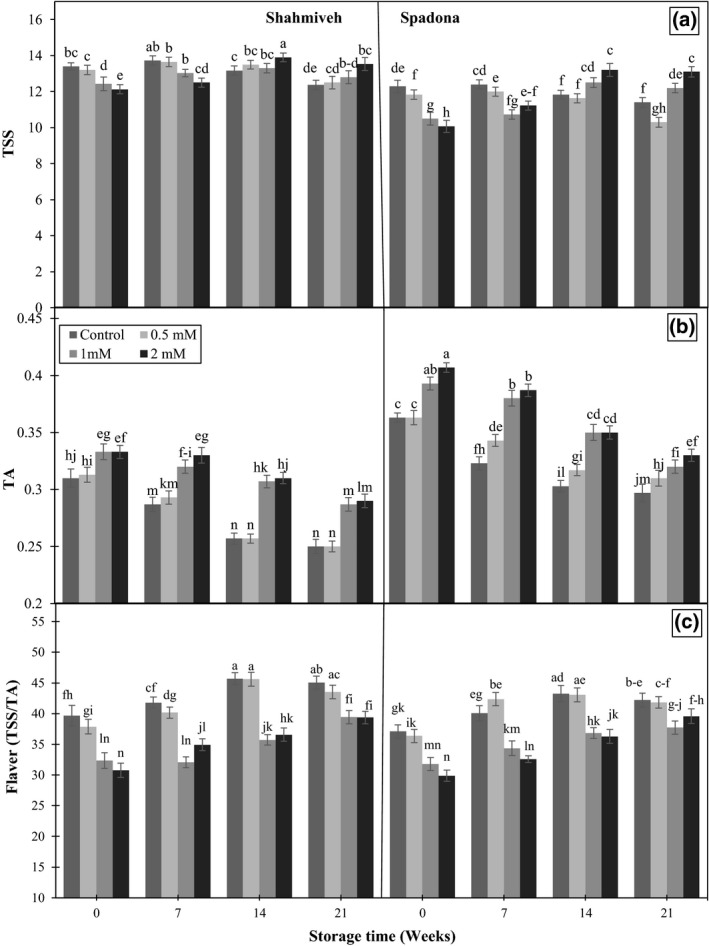
Effect of putrescine on total soluble solids (TSS) (a), titratable acidity (TA) (b), and flavor index (c) of pear cultivars of “Shah‐Miveh” and “Spadona” stored at 0 ± 1°C. Data are means ± *SE*. Values with similar letters did not show significant difference (*p* < 0.05)

Irrespective of treatments, an increase in the level of TSS and decrease in TA were observed which accounted for the higher flavor index in all samples at harvest as well as prolonged storage (Figure 2). Although higher level of TA and lower level of TSS was detected in “Spadona” than “Shah‐Miveh,” putrescine treatment at concentrations of 1 and 2 mM better maintained TSS, TA, and subsequently flavor index on the contrary to the lowest concentration (0.5 mM) in both cultivars (Figure 2a,b). The effect of putrescine on retarding degradation of TSS and retaining TA is ascribed to reduction in respiration (Valero et al., 2002) and ethylene synthesis (Barman, Ram, & Pal, 2011; Zokaee Khosroshahi, Esna‐Ashari, & Ershadi, 2007). Similar results were reported in grape (Mirdehghan & Rahimi, 2016) and mango (Razzaq, Khan, Malik, Shahid, & Ullah, 2014).

### 3.3 Color

**Figure 3:**
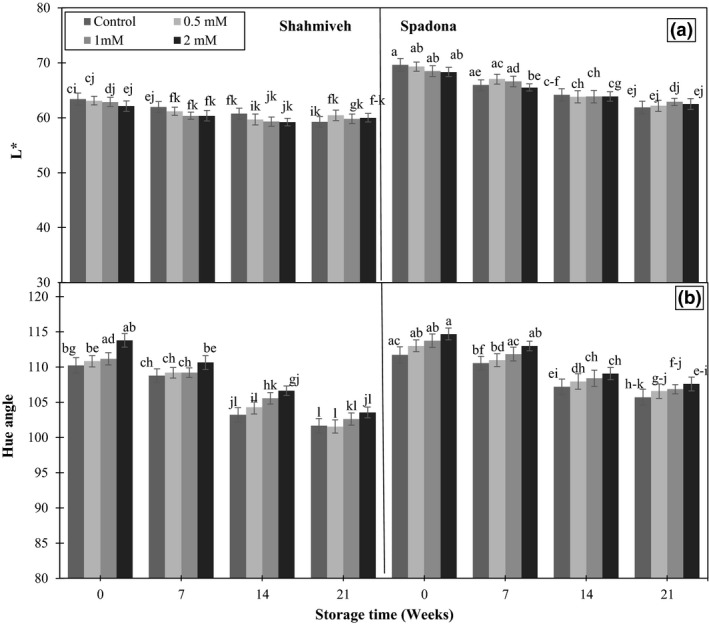
Effect of putrescine on L* (a) and hue angle (b) of pear cultivars of “Shah‐Miveh” and “Spadona” stored at 0 ± 1°C. Data are means ± *SE*. Values with similar letters did not show significant difference (*p* < 0.05)

L* and hue angle decreased throughout the storage period in all samples, although L* was higher in “Spadona” while hue angle showed the reverse trend compared to “Shah‐Miveh” (Figure 3a,b). Decrease in hue angle is characteristic ripening development and change of color from green to yellow which was happened by degradation of chlorophyll and carotenoids synthesis during storage. Putrescine treatment at higher values effectively suppressed color changes in both cultivars than control at harvest and during storage. The positive effect of putrescine treatment on inhibition of color change is due to suppression of rates of respiration and ethylene production and consequently delays of senescence (Drake & Chen, 2000).

### 3.4 Ascorbic acid

**Figure 4:**
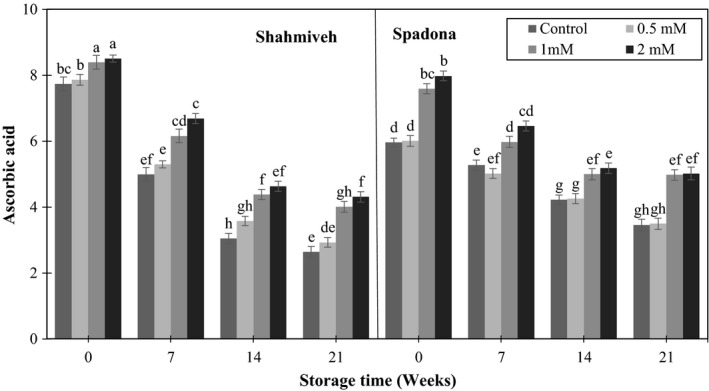
Effect of putrescine on ascorbic acid of pear cultivars of “Shah‐Miveh” and “Spadona” stored at 0 ± 1°C. Data are means ± *SE*. Values with similar letters did not show significant difference (*p* < 0.05)

Ascorbic acid decreased in all treated and untreated fruits as the storage time progressed. This trend was higher in “Shah‐Miveh” than “Spadona.” The highest level of ascorbic acid was observed in putrescine 1 and 2 mM in both cultivars compared to control immediately after harvest and different sampling dates (Figure 4). Declined ascorbic acid could be due to conversion of dehydroascobic to diketogulonic acid by oxidation. The effect of putrescine treatment to maintain ascorbic acid content may be ascribed to decreased or delayed ascorbate oxidase activity (Ishaq, Rathore, Majeed, Awan, & Zulfiqar‐Ali‐Shah, 2009). Similarly, prestorage application of putrescine retained ascorbic acid in apricot (Davarynejad, Zarei, Ardakani, & Nasrabadi, 2013).

### 3.5 Total phenol and total antioxidant activity

**Figure 5:**
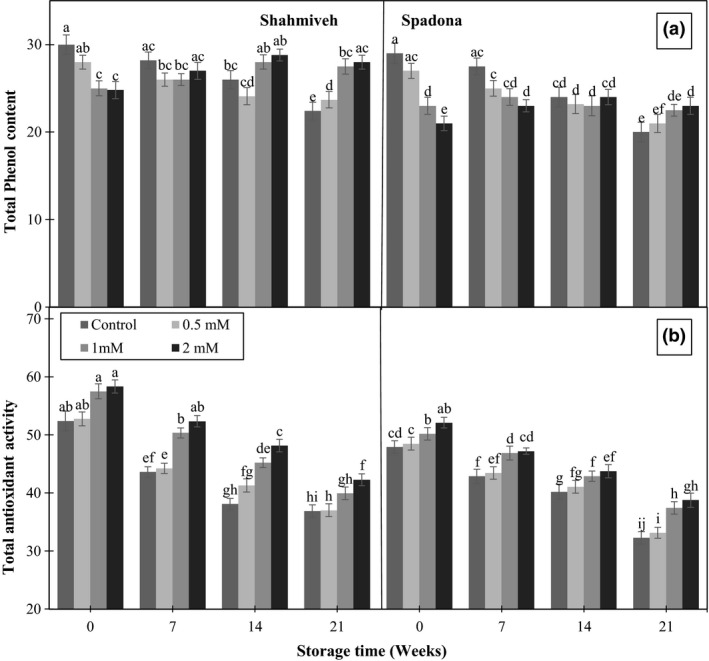
Effect of putrescine on total phenol content (a) and total antioxidant activity (b) of pear cultivars of “Shah‐Miveh” and “Spadona” stored at 0 ± 1°C. Data are means ± *SE*. Values with similar letters did not show significant difference (*p* < 0.05)

Total phenol and TAA levels declined as the storage time progressed, while they significantly influenced by putrescine treatment, in which the values of them in fruits treated with 1 and 2 mM putrescine were higher than those in control along the cold storage. However, the effect of treatments was depended on cultivars as well. In “Shah‐Miveh” higher levels of putrescine were more effective than 0.5 mM in terms of retarding TP along with TAA degradation throughout the storage (Figure 5). The role of putrescine treatment in maintenance of TP and TAA could be ascribed to a delay in senescence process (Arora, Sairam, & Srivastava, 2002; Razzaq et al., 2014).

Moreover, it is suggested that TP content is directly correlated with TAA. A positive correlation between TP and TAA has been reported in apricot (Ghasemnezhad, Shiri, & Sanavi, 2010) and mango (Palafox‐Carlos et al., 2012).

### 3.6 Microbial activity

**Figure 6:**
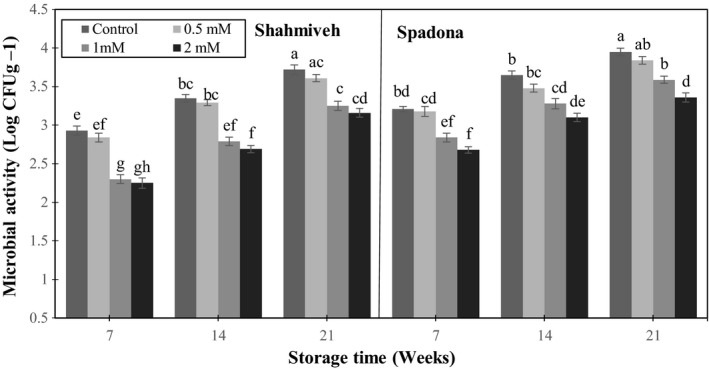
Effect of putrescine on microbial activity of pear cultivars of “Shah‐Miveh” and “Spadona” stored at 0 ± 1°C. Data are means ± *SE*. Values with similar letters did not show significant difference (*p* < 0.05)

Preharvest application of pear fruits with putrescine significantly reduced the microbial activity of both cultivars along the storage. At the end of storage, putrescine 2 mM was known as the most effective treatment on controlling microbial activity of stored pears (Figure 6). In pear, mechanical injury of fruits leads to fungal pathogens development during handling and storage. It is well known that *Penicillium expansum* is one of the most important fungi which leads to blue mold and SL limitation during storage and marketing. The results indicate an increase in fungal infection of pears during storage while that was significantly lower in 1 and 2 mM putrescine‐treated fruits. Mirdehghan and Rahimi (2016) studied the use of polyamines on table grape cv. “Rishbaba” and “Olhoghi.” They reported putrescine‐treated fruits maintained less fungal infection symptoms along with a good appearance than control fruits. Therefore polyamines may have all requirements of an alternative approach for management of postharvest decay (Romanazzi, Lichter, Mlikota Gabler, & Smilanick, 2012).

## 4 CONCLUDING REMARKS

Weight loss, softening, and fungal infection increased along the storage while the rate of them significantly was delayed in fruits treated with 1 and 2 mM. Besides, higher values of putrescine reduced the degradation rates of TP content and TAA during cold storage. Furthermore, at the end of storage, putrescine treatment at 1 and 2 mM showed lower changes in TSS, TA, flavor index, and skin color on the contrary to control.

## CONFLICT OF INTEREST

The authors declare no conflict of interest.
